# Supplementary material for: Dental Pain Medication Prescriptions in Minas Gerais, Brazil (2011–2021): A Time-Series Analysis
Source: Int J Environ Res Public Health. 2023 Sep 21;20(18):6795. doi: 10.3390/ijerph20186795 (PMC10531368; doi:10.3390/ijerph20186795)
Supplement: Supplementary file 1 [file ijerph-20-06795-s001.zip › ijerph-2597473-supplementary.pdf]

Table S1. Adjustment of the linear time-series regression model of the average log of 1DDD/1,000 inhabit-ants/year of NSAIDS.

```
Call:
lm(formula = log(DDD) ~ residuosdef2 + residuosdef3 + residuosdef4 +
    tt + City + tt:City, data = dt)

Residuals:
    Min      1Q  Median      3Q     Max
-3.5528 -0.3125  0.0243  0.4058  2.7125

Coefficients:
                                    Estimate     Std. Error t value Pr(>|t|)
(Intercept)                          1.94837    0.57271   3.402 0.000749 ***
residuosdef2                        -0.20938    0.05566  -3.762 0.000198 ***
residuosdef3                        -0.29904    0.05782  -5.172 3.98e-07 ***
residuosdef4                        -0.33560    0.06521  -5.146 4.51e-07 ***
tt                                   0.37985    0.08448   4.497 9.50e-06 ***
City ANTONIO CARLOS                  0.12481    0.80953   0.154 0.877567
City ARAPUA                          0.51681    0.81006   0.638 0.523917
City BALDIM                          2.49191    0.81124   3.072 0.002301 **
City BELA VISTA DE MINAS             2.88497    0.80952   3.564 0.000418 ***
City BONFINOPOLIS DE MINAS           2.79380    0.81034   3.448 0.000637 ***
City CATAS ALTAS                     3.93701    0.81003   4.860 1.80e-06 ***
City CONEGO MARINHO                  0.75728    0.80962   0.935 0.350277
City CONGONHAS DO NORTE              3.43479    0.81040   4.238 2.91e-05 ***
City DOM CAVATI                      0.75769    0.81485   0.930 0.353107
City DOURADOQUARA                    3.57622    0.81082   4.411 1.39e-05 ***
City INDIANOPOLIS                    2.95797    0.80968   3.653 0.000300 ***
City JEQUITIBA                      -0.54795    0.80984  -0.677 0.499114
City JOANESIA                        2.80149    0.80999   3.459 0.000612 ***
City JOAO MONLEVADE                 -3.08393    0.81017  -3.807 0.000167 ***
City LAGOA FORMOSA                   2.08493    0.80981   2.575 0.010460 *
City LIMEIRA DO OESTE                1.58685    0.80953   1.960 0.050790 .
City MALACACHETA                    -0.92290    0.80971  -1.140 0.255179
City MESQUITA                        0.90947    0.80987   1.123 0.262242
City MONJOLOS                        3.07809    0.81075   3.797 0.000174 ***
City NOVA PONTE                      1.69089    0.81046   2.086 0.037695 *
City PALMA                           2.99534    0.80956   3.700 0.000252 ***
City PEDRA DO INDAIA                 3.45909    0.81005   4.270 2.54e-05 ***
City PRESIDENTE JUSCELINO            0.67182    0.80954   0.830 0.407196
City RIO PARANAIBA                  -1.67014    0.81020  -2.061 0.040027 *
City SANTA BARBARA                  -2.85477    0.81074  -3.521 0.000488 ***
City SAO DOMINGOS DO PRATA          -1.94685    0.80973  -2.404 0.016738 *
City SAO GONCALO DO ABAETE           2.53549    0.81532   3.110 0.002031 **
City SAO GONCALO DO RIO ABAIXO       4.43388    0.80959   5.477 8.46e-08 ***
City SAO JOAO DO MANHUACU            1.31146    0.81025   1.619 0.106465
City SAO JOSE DA SAFIRA              0.99156    0.80956   1.225 0.221496
City SAO SEBASTIAO DO RIO PRETO      3.71608    0.80998   4.588 6.31e-06 ***
City SAO TOMAS DE AQUINO             2.06573    0.81020   2.550 0.011223 *
City SAO VICENTE DE MINAS            2.48386    0.80981   3.067 0.002334 **
City SENHORA DOS REMEDIOS            2.53470    0.80950   3.131 0.001892 **
City SETE LAGOAS                    -6.82214    0.81313  -8.390 1.33e-15 ***
City UBERABA                        -4.58765    0.80955  -5.667 3.11e-08 ***
City VERISSIMO                       2.31805    0.80999   2.862 0.004474 **
tt:City ANTONIO CARLOS              -0.06978    0.11937  -0.585 0.559230
tt:City ARAPUA                      -0.07960    0.11963  -0.665 0.506243
tt:City BALDIM                      -0.35328    0.11981  -2.949 0.003412 **
tt:City BELA VISTA DE MINAS         -0.29520    0.11937  -2.473 0.013887 *
tt:City BONFINOPOLIS DE MINAS       -0.42843    0.11963  -3.581 0.000392 ***
tt:City CATAS ALTAS                 -0.38833    0.11942  -3.252 0.001262 **
tt:City CONEGO MARINHO              -0.21114    0.11942  -1.768 0.077953 .
tt:City CONGONHAS DO NORTE          -0.53999    0.11960  -4.515 8.75e-06 ***
tt:City DOM CAVATI                  -0.15515    0.12088  -1.283 0.200194
tt:City DOURADOQUARA                -0.44722    0.11969  -3.737 0.000219 ***
tt:City INDIANOPOLIS                -0.27411    0.11937  -2.296 0.022272 *
tt:City JEQUITIBA                   -0.13337    0.11952  -1.116 0.265261
tt:City JOANESIA                    -0.43692    0.11953  -3.655 0.000297 ***
tt:City JOAO MONLEVADE              -0.06292    0.11942  -0.527 0.598604
tt:City LAGOA FORMOSA               -0.16622    0.11942  -1.392 0.164891
tt:City LIMEIRA DO OESTE            -0.21875    0.11937  -1.833 0.067748 .
tt:City MALACACHETA                 -0.09109    0.11944  -0.763 0.446217
tt:City MESQUITA                    -0.23589    0.11951  -1.974 0.049214 *
tt:City MONJOLOS                    -0.39060    0.11985  -3.259 0.001231 **
tt:City NOVA PONTE                  -0.12145    0.11972  -1.014 0.311124
tt:City PALMA                       -0.70958    0.11940  -5.943 6.96e-09 ***
tt:City PEDRA DO INDAIA             -0.37368    0.11949  -3.127 0.001918 **
tt:City PRESIDENTE JUSCELINO        -0.13938    0.11937  -1.168 0.243787
tt:City RIO PARANAIBA               -0.16926    0.11956  -1.416 0.157778
tt:City SANTA BARBARA               -0.31533    0.11959  -2.637 0.008758 **
tt:City SAO DOMINGOS DO PRATA       -0.08668    0.11944  -0.726 0.468494
tt:City SAO GONCALO DO ABAETE       -0.51436    0.12021  -4.279 2.45e-05 ***
tt:City SAO GONCALO DO RIO ABAIXO   -0.53819    0.11940  -4.508 9.04e-06 ***
tt:City SAO JOAO DO MANHUACU        -0.48781    0.11959  -4.079 5.64e-05 ***
tt:City SAO JOSE DA SAFIRA          -0.30396    0.11939  -2.546 0.011341 *
tt:City SAO SEBASTIAO DO RIO PRETO -0.36481    0.11943  -3.055 0.002433 **
```

```
tt:CitySAO TOMAS DE AQUINO        -0.51641    0.11957  -4.319 2.06e-05 ***
tt:CitySAO VICENTE DE MINAS       -0.31418    0.11941  -2.631 0.008899 **
tt:CitySENHORA DOS REMEDIOS       -0.44881    0.11935  -3.760 0.000200 ***
tt:CitySETE LAGOAS                 0.36744    0.12072   3.044 0.002518 **
tt:CityUBERABA                    -0.25704    0.11938  -2.153 0.032011 *
tt:CityVERISSIMO                  -0.19863    0.11942  -1.663 0.097177 .
---
Signif. codes:  0 '***' 0.001 '**' 0.01 '*' 0.05 '.' 0.1 ' ' 1

Residual standard error: 0.8851 on 339 degrees of freedom
Multiple R-squared:  0.8567,     Adjusted R-squared:  0.8237
F-statistic: 25.97 on 78 and 339 DF,  p-value: < 2.2e-16
```

Table S2. Full linear time-series regression model for DDD/1,000 inhabitants/year of NSAIDS and covariates.

```
> model3=lm(log(DDD)~Coverage+FDA+Ext+Endo+DP+tt+City+tt:City,data=dt) #
> summary(model3)  # Adjust the least squares model.

Call:
lm(formula = log(DDD) ~ Coverage + FDA + Ext + Endo + DP + tt +
    City + tt:City, data = dt)

Residuals:
    Min      1Q  Median      3Q     Max
-3.6242 -0.3586  0.0493  0.4513  2.6633

Coefficients:
                                    Estimate Std. Error  tvalue  Pr(>|t|)
(Intercept)                         1.669e+00  7.723e-01   2.161 0.031396 *
Coverage                            1.612e-03  4.802e-03   0.336 0.737347
FDA                                -1.161e-05  7.583e-05  -0.153 0.878454
Ext                                 8.109e-03  9.064e-03   0.895 0.371623
Endo                               -3.613e-03  8.931e-03  -0.405 0.686067
DP                                  4.539e-03  2.061e-03   2.202 0.028359 *
tt                                  3.253e-01  9.010e-02   3.611 0.000352 ***
City ANTONIO CARLOS                 1.950e-01  8.573e-01   0.227 0.820177
City ARAPUA                         3.759e-01  8.656e-01   0.434 0.664335
City BALDIM                         1.686e+00  8.751e-01   1.926 0.054898 .
City BELA VISTA DE MINAS            3.049e+00  9.182e-01   3.321 0.000997 ***
City BONFINOPOLIS DE MINAS          2.170e+00  8.639e-01   2.512 0.012488 *
City CATAS ALTAS                    3.617e+00  8.810e-01   4.106 5.06e-05 ***
City CONEGO MARINHO                 1.055e-02  1.003e+00   0.011 0.991607
City CONGONHAS DO NORTE             2.691e+00  8.952e-01   3.006 0.002844 **
City DOM CAVATI                    -8.007e-01  9.017e-01  -0.888 0.375156
City DOURADOQUARA                   2.519e+00  8.799e-01   2.863 0.004456 **
City INDIANOPOLIS                   3.077e+00  8.579e-01   3.587 0.000384 ***
City JEQUITIBA                     -7.543e-01  8.779e-01  -0.859 0.390850
City JOANESIA                       2.828e+00  8.569e-01   3.300 0.001069 **
City JOAO MONLEVADE                -3.523e+00  9.570e-01  -3.681 0.000270 ***
City LAGOA FORMOSA                  1.490e+00  8.842e-01   1.685 0.092888 .
City LIMEIRA DO OESTE               1.233e+00  8.637e-01   1.428 0.154263
City MALACACHETA                   -1.161e+00  8.646e-01  -1.343 0.180208
City MESQUITA                       7.642e-01  8.565e-01   0.892 0.372913
City MONJOLOS                       1.637e+00  9.152e-01   1.789 0.074527 .
City NOVA PONTE                     1.605e+00  8.636e-01   1.859 0.063919 .
City PALMA                          2.872e+00  8.576e-01   3.349 0.000904 ***
City PEDRA DO INDAIA                3.086e+00  8.628e-01   3.576 0.000399 ***
City PRESIDENTE JUSCELINO          -1.243e-01  8.946e-01  -0.139 0.889557
City RIO PARANAIBA                 -1.839e+00  8.701e-01  -2.114 0.035274 *
City SANTA BARBARA                 -3.265e+00  8.977e-01  -3.637 0.000319 ***
City SAO DOMINGOS DO PRATA         -2.293e+00  8.698e-01  -2.637 0.008761 **
City SAO GONCALO DO ABAETE          2.033e+00  9.112e-01   2.231 0.026331 *
City SAO GONCALO DO RIO ABAIXO      4.156e+00  8.738e-01   4.757 2.92e-06 ***
City SAO JOAO DO MANHUACU           7.250e-01  8.681e-01   0.835 0.404236
City SAO JOSE DA SAFIRA             6.781e-01  8.675e-01   0.782 0.434964
City SAO SEBASTIAO DO RIO PRETO     3.222e+00  1.132e+00   2.846 0.004691 **
City SAO TOMAS DE AQUINO            1.968e+00  8.573e-01   2.296 0.022295 *
City SAO VICENTE DE MINAS           2.144e+00  8.633e-01   2.484 0.013490 *
City SENHORA DOS REMEDIOS           2.483e+00  8.815e-01   2.817 0.005136 **
City SETE LAGOAS                   -7.250e+00  9.214e-01  -7.869 4.93e-14 ***
City UBERABA                       -4.632e+00  8.689e-01  -5.330 1.80e-07 ***
City VERISSIMO                      1.960e+00  8.691e-01   2.255 0.024747 *
tt:City ANTONIO CARLOS             -5.970e-02  1.263e-01  -0.473 0.636802
tt:City ARAPUA                     -5.540e-02  1.277e-01  -0.434 0.664641
tt:City BALDIM                     -2.309e-01  1.276e-01  -1.809 0.071379 .
tt:City BELA VISTA DE MINAS        -2.729e-01  1.284e-01  -2.125 0.034320 *
tt:City BONFINOPOLIS DE MINAS      -3.199e-01  1.272e-01  -2.514 0.012394 *
tt:City CATAS ALTAS                -3.697e-01  1.273e-01  -2.906 0.003908 **
tt:City CONEGO MARINHO             -1.047e-01  1.464e-01  -0.715 0.475337
tt:City CONGONHAS DO NORTE         -4.175e-01  1.290e-01  -3.238 0.001324 **
tt:City DOM CAVATI                  1.122e-01  1.325e-01   0.847 0.397739
tt:City DOURADOQUARA               -2.919e-01  1.289e-01  -2.265 0.024146 *
tt:City INDIANOPOLIS               -2.785e-01  1.263e-01  -2.206 0.028065 *
tt:City JEQUITIBA                  -1.203e-01  1.280e-01  -0.940 0.347976
tt:City JOANESIA                   -4.222e-01  1.263e-01  -3.342 0.000926 ***
tt:City JOAO MONLEVADE              4.224e-02  1.297e-01   0.326 0.744982
tt:City LAGOA FORMOSA              -7.789e-02  1.320e-01  -0.590 0.555579
tt:City LIMEIRA DO OESTE           -1.371e-01  1.276e-01  -1.074 0.283502
tt:City MALACACHETA                -1.135e-01  1.265e-01  -0.897 0.370120
tt:City MESQUITA                   -1.698e-01  1.264e-01  -1.344 0.179949
tt:City MONJOLOS                   -1.941e-01  1.304e-01  -1.488 0.137705
tt:City NOVA PONTE                 -1.196e-01  1.278e-01  -0.936 0.350181
tt:City PALMA                      -6.411e-01  1.268e-01  -5.057 7.00e-07 ***
tt:City PEDRA DO INDAIA            -3.537e-01  1.400e-01  -2.527 0.011975 *
tt:City PRESIDENTE JUSCELINO       -9.671e-02  1.308e-01  -0.739 0.460129
tt:City RIO PARANAIBA              -1.027e-01  1.265e-01  -0.812 0.417261
tt:City SANTA BARBARA              -2.675e-01  1.466e-01  -1.825 0.068874 .
tt:City SAO DOMINGOS DO PRATA      -2.921e-02  1.274e-01  -0.229 0.818791
```

```
tt:City SAO GONCALO DO ABAETE      -3.983e-01  1.300e-01  -3.065 0.002352 **
tt:City SAO GONCALO DO RIO ABAIXO  -5.214e-01  1.339e-01  -3.894 0.000119 ***
tt:City SAO JOAO DO MANHUACU       -3.647e-01  1.277e-01  -2.856 0.004556 **
tt:City SAO JOSE DA SAFIRA         -2.557e-01  1.282e-01  -1.995 0.046873 *
tt:City SAO SEBASTIAO DO RIO PRETO -3.038e-01  1.425e-01  -2.131 0.033788 *
tt:City SAO TOMAS DE AQUINO        -4.503e-01  1.266e-01  -3.557 0.000429 ***
tt:City SAO VICENTE DE MINAS       -2.435e-01  1.290e-01  -1.888 0.059883 .
tt:City SENHORA DOS REMEDIOS       -4.137e-01  1.289e-01  -3.210 0.001455 **
tt:City SETE LAGOAS                 5.391e-01  1.270e-01   4.244 2.84e-05 ***
tt:City UBERABA                    -1.943e-01  1.270e-01  -1.530 0.127071
tt:City VERISSIMO                  -1.524e-01  1.280e-01  -1.190 0.234766
---
Signif. codes:  0 ‘***’ 0.001 ‘**’ 0.01 ‘*’ 0.05 ‘.’ 0.1 ‘ ’ 1

Residual standard error: 0.9358 on 337 degrees of freedom
Multiple R-squared:  0.8407,          Adjusted R-squared:  0.8029
F-statistic: 22.23 on 80 and 337 DF,  p-value: < 2.2e-16
```

Table S3. Final linear time-series regression model for DDD/1,000 inhabitants/year of NSAIDS and covariates.

```
Call:
lm(formula = log(DDD) ~ DP + tt + City + tt:City, data = dt)

Residuals:
    Min      1Q  Median      3Q     Max
-3.8190 -0.3726  0.0529  0.4284  2.7604

Coefficients:
                                   Estimate    Std. Error tvalue Pr(>|t|)
(Intercept)                         2.036835   0.609189   3.344 0.000919 ***
DP                                  0.004755   0.002027   2.346 0.019566 *
tt                                  0.327170   0.090617   3.610 0.000352 ***
City ANTONIO CARLOS                 0.234142   0.861493   0.272 0.785951
City ARAPUA                         0.731905   0.861510   0.850 0.396165
City BALDIM                         2.195972   0.861436   2.549 0.011234 *
City BELA VISTA DE MINAS            2.948325   0.861278   3.423 0.000694 ***
City BONFINOPOLIS DE MINAS          2.490708   0.861459   2.891 0.004083 **
City CATAS ALTAS                    3.606916   0.863333   4.178 3.74e-05 ***
City CONEGO MARINHO                 0.716633   0.861345   0.832 0.405995
City CONGONHAS DO NORTE             2.893657   0.870172   3.325 0.000979 ***
City DOM CAVATI                    -0.049152   0.863095  -0.057 0.954620
City DOURADOQUARA                   2.940355   0.871007   3.376 0.000821 ***
City INDIANOPOLIS                   2.988696   0.862369   3.466 0.000596 ***
City JEQUITIBA                     -0.259641   0.863979  -0.301 0.763965
City JOANESIA                       2.825208   0.861767   3.278 0.001152 **
City JOAO MONLEVADE                -3.185030   0.861503  -3.697 0.000254 ***
City LAGOA FORMOSA                  1.827029   0.879616   2.077 0.038543 *
City LIMEIRA DO OESTE               1.551052   0.861225   1.801 0.072589 .
City MALACACHETA                   -0.997454   0.865180  -1.153 0.249764
City MESQUITA                       0.794441   0.861596   0.922 0.357150
City MONJOLOS                       2.187479   0.895179   2.444 0.015047 *
City NOVA PONTE                     1.848724   0.862753   2.143 0.032835 *
City PALMA                          3.008536   0.861641   3.492 0.000543 ***
City PEDRA DO INDAIA                3.311803   0.861609   3.844 0.000145 ***
City PRESIDENTE JUSCELINO           0.590530   0.861616   0.685 0.493573
City RIO PARANAIBA                 -1.875760   0.861337  -2.178 0.030111 *
City SANTA BARBARA                 -3.400207   0.866644  -3.923 0.000106 ***
City SAO DOMINGOS DO PRATA         -2.072593   0.861667  -2.405 0.016690 *
City SAO GONCALO DO ABAETE          1.987237   0.861810   2.306 0.021716 *
City SAO GONCALO DO RIO ABAIXO      4.196084   0.867618   4.836 2.01e-06 ***
City SAO JOAO DO MANHUACU           1.116163   0.861560   1.296 0.196019
City SAO JOSE DA SAFIRA             0.780837   0.868363   0.899 0.369177
City SAO SEBASTIAO DO RIO PRETO     3.331971   0.865842   3.848 0.000142 ***
City SAO TOMAS DE AQUINO            1.906484   0.861927   2.212 0.027637 *
City SAO VICENTE DE MINAS           2.407357   0.861667   2.794 0.005503 **
City SENHORA DOS REMEDIOS           2.576464   0.861492   2.991 0.002986 **
City SETE LAGOAS                   -7.347054   0.861318  -8.530 4.85e-16 ***
City UBERABA                       -4.732710   0.861876  -5.491 7.81e-08 ***
City VERISSIMO                      2.303424   0.865747   2.661 0.008168 **
tt:City ANTONIO CARLOS             -0.073017   0.127001  -0.575 0.565716
tt:City ARAPUA                     -0.103929   0.127219  -0.817 0.414536
tt:City BALDIM                     -0.283006   0.127024  -2.228 0.026534 *
tt:City BELA VISTA DE MINAS        -0.278132   0.127565  -2.180 0.029918 *
tt:City BONFINOPOLIS DE MINAS      -0.348008   0.127553  -2.728 0.006695 **
tt:City CATAS ALTAS                -0.363291   0.126976  -2.861 0.004482 **
tt:City CONEGO MARINHO             -0.196026   0.126984  -1.544 0.123588
tt:City CONGONHAS DO NORTE         -0.448685   0.128556  -3.490 0.000546 ***
tt:City DOM CAVATI                  0.005146   0.127367   0.040 0.967798
tt:City DOURADOQUARA               -0.340073   0.128358  -2.649 0.008439 **
tt:City INDIANOPOLIS               -0.280615   0.127042  -2.209 0.027851 *
tt:City JEQUITIBA                  -0.179210   0.127071  -1.410 0.159361
tt:City JOANESIA                   -0.437179   0.127002  -3.442 0.000649 ***
tt:City JOAO MONLEVADE             -0.028695   0.127402  -0.225 0.821936
tt:City LAGOA FORMOSA              -0.134768   0.129613  -1.040 0.299184
tt:City LIMEIRA DO OESTE           -0.178794   0.127487  -1.402 0.161692
tt:City MALACACHETA                -0.125515   0.126997  -0.988 0.323694
tt:City MESQUITA                   -0.178687   0.127102  -1.406 0.160679
tt:City MONJOLOS                   -0.266074   0.128443  -2.072 0.039060 *
tt:City NOVA PONTE                 -0.157224   0.127732  -1.231 0.219213
tt:City PALMA                      -0.668557   0.127162  -5.258 2.58e-07 ***
tt:City PEDRA DO INDAIA            -0.372329   0.128024  -2.908 0.003873 **
tt:City PRESIDENTE JUSCELINO       -0.180205   0.128111  -1.407 0.160446
tt:City RIO PARANAIBA              -0.108314   0.127054  -0.852 0.394535
tt:City SANTA BARBARA              -0.228805   0.127703  -1.792 0.074068 .
tt:City SAO DOMINGOS DO PRATA      -0.074390   0.127075  -0.585 0.558663
tt:City SAO GONCALO DO ABAETE      -0.408731   0.127125  -3.215 0.001428 **
tt:City SAO GONCALO DO RIO ABAIXO  -0.521267   0.127511  -4.088 5.43e-05 ***
tt:City SAO JOAO DO MANHUACU       -0.413530   0.127265  -3.249 0.001272 **
```

```
tt:City SAO JOSE DA SAFIRA         -0.275715   0.128299  -2.149 0.032337 *
tt:City SAO SEBASTIAO DO RIO PRETO -0.309457   0.127633  -2.425 0.015845 *
tt:City SAO TOMAS DE AQUINO        -0.453954   0.127067  -3.573 0.000404 ***
tt:City SAO VICENTE DE MINAS       -0.296898   0.126988  -2.338 0.019965 *
tt:City SENHORA DOS REMEDIOS       -0.437191   0.127102  -3.440 0.000655 ***
tt:City SETE LAGOAS                 0.521896   0.127353   4.098 5.21e-05 ***
tt:City UBERABA                    -0.210626   0.127671  -1.650 0.099913 .
tt:City VERISSIMO                  -0.182364   0.128403  -1.420 0.156449
---
Signif. codes:  0 '***' 0.001 '**' 0.01 '*' 0.05 '.' 0.1 ' ' 1

Residual standard error: 0.9417 on 341 degrees of freedom
Multiple R-squared:  0.8368,          Adjusted R-squared:  0.8004
F-statistic: 23.01 on 76 and 341 DF,  p-value: < 2.2e-16
```

Table S4. Adjustment of the linear time-series regression model for log of DDD/1,000 inhabitants/year of analgesics.

```
Call:
lm(formula = log(DDD) ~ residuosdef2 + residuosdef3 + residuosdef4 +
    tt + City + tt:City, data = dt)

Residuals:
    Min      1Q  Median      3Q     Max
-3.4230 -0.3441  0.0224  0.4296  2.6927

Coefficients:
                                    Estimate     Std. Error   t value Pr(>|t|)
(Intercept)                          0.4587646  0.5183599   0.885 0.376694
residuosdef2                        -0.2738633  0.0536506  -5.105 5.23e-07 ***
residuosdef3                        -0.2059644  0.0551056  -3.738 0.000214 ***
residuosdef4                        -0.2917379  0.0608577  -4.794 2.34e-06 ***
tt                                   0.2816742  0.0764311   3.685 0.000261 ***
City ARAPUA                          0.5660692  0.7330887   0.772 0.440489
City BALDIM                          2.5469238  0.7337128   3.471 0.000577 ***
City BELA VISTA DE MINAS             2.4355222  0.7333397   3.321 0.000982 ***
City BONFINOPOLIS DE MINAS           2.9054751  0.7331366   3.963 8.82e-05 ***
City CARNEIRINHO                    -1.8183032  0.7335968  -2.479 0.013618 *
City CATAS ALTAS                     4.1410321  0.7344414   5.638 3.33e-08 ***
City CLAUDIO                        -0.8360687  0.7379285  -1.133 0.257924
City CONEGO MARINHO                  1.4714822  0.7344899   2.003 0.045836 *
City CONGONHAS DO NORTE              3.0305208  0.7334280   4.132 4.42e-05 ***
City CONQUISTA                       1.5560284  0.7331157   2.122 0.034436 *
City DOM BOSCO                       0.9346940  0.7359268   1.270 0.204821
City DOM CAVATI                      1.1909739  0.7340504   1.622 0.105524
City DOM SILVERIO                    0.2876546  0.7339773   0.392 0.695341
City DOURADOQUARA                    3.6854934  0.7331361   5.027 7.66e-07 ***
City INDIANOPOLIS                    3.5516985  0.7331627   4.844 1.85e-06 ***
City JABOTICATUBAS                  -3.3285691  0.7344928  -4.532 7.82e-06 ***
City JEQUITIBA                       0.6998710  0.7330917   0.955 0.340338
City JOANESIA                        2.7926220  0.7334619   3.807 0.000163 ***
City JOAO MONLEVADE                 -4.0662682  0.7331116  -5.547 5.43e-08 ***
City LAGOA FORMOSA                   2.2933578  0.7335628   3.126 0.001905 **
City LIMEIRA DO OESTE                3.1426582  0.7331130   4.287 2.30e-05 ***
City MALACACHETA                     0.2792817  0.7340753   0.380 0.703819
City MESQUITA                        1.0148051  0.7339674   1.383 0.167582
City MINDURI                         0.3347115  0.7336140   0.456 0.648468
City MONJOLOS                        3.7450471  0.7336099   5.105 5.22e-07 ***
City NOVA PONTE                      0.8333061  0.7332864   1.136 0.256498
City PALMA                           3.7014001  0.7334416   5.047 6.96e-07 ***
City PATOS DE MINAS                 -4.9168540  0.7342305  -6.697 7.60e-11 ***
City PEDRA DO INDAIA                 3.3457888  0.7330873   4.564 6.77e-06 ***
City PRESIDENTE JUSCELINO            0.9059260  0.7335319   1.235 0.217579
City RIO PIRACICABA                 -2.1585867  0.7332011  -2.944 0.003436 **
City SANTO ANTONIO DO MONTE         -3.2572018  0.7338013  -4.439 1.18e-05 ***
City SAO GONCALO DO ABAETE           1.2484795  0.7344424   1.700 0.089959 .
City SAO GONCALO DO RIO ABAIXO       4.2047979  0.7333652   5.734 1.99e-08 ***
City SAO JOAO DO MANHUACU            1.6130777  0.7343509   2.197 0.028646 *
City SAO JOSE DA SAFIRA              2.4188086  0.7331552   3.299 0.001060 **
City SAO SEBASTIAO DO RIO PRETO      4.4489227  0.7331437   6.068 3.10e-09 ***
City SAO TOMAS DE AQUINO             1.1801965  0.7334059   1.609 0.108394
City SAO VICENTE DE MINAS            1.9059871  0.7333011   2.599 0.009705 **
City SENHORA DOS REMEDIOS            2.6979398  0.7332588   3.679 0.000267 ***
City UBERABA                        -4.9818594  0.7336532  -6.790 4.26e-11 ***
City VERISSIMO                       2.0551683  0.7344137   2.798 0.005395 **
tt:City ARAPUA                       0.0001513  0.1080955   0.001 0.998884
tt:City BALDIM                      -0.1887476  0.1082071  -1.744 0.081904 .
tt:City BELA VISTA DE MINAS         -0.1356757  0.1081771  -1.254 0.210532
tt:City BONFINOPOLIS DE MINAS       -0.2184619  0.1081022  -2.021 0.043985 *
tt:City CARNEIRINHO                  0.2845618  0.1082877   2.628 0.008938 **
tt:City CATAS ALTAS                 -0.2921835  0.1083575  -2.696 0.007316 **
tt:City CLAUDIO                      0.0981477  0.1093523   0.898 0.369995
tt:City CONEGO MARINHO              -0.1825408  0.1082855  -1.686 0.092659 .
tt:City CONGONHAS DO NORTE          -0.4035550  0.1081751  -3.731 0.000220 ***
tt:City CONQUISTA                   -0.4149985  0.1080976  -3.839 0.000144 ***
tt:City DOM BOSCO                    0.0756348  0.1089636   0.694 0.488021
```

```
tt:City DOM CAVATI                     0.0130967  0.1084850   0.121 0.903973
tt:City DOM SILVERIO                   0.0607687  0.1084099   0.561 0.575434
tt:City DOURADOQUARA                  -0.3032056  0.1081047  -2.805 0.005292 **
tt:City INDIANOPOLIS                  -0.2142150  0.1081297  -1.981 0.048294 *
tt:City JABOTICATUBAS                  0.2818154  0.1085331   2.597 0.009777 **
tt:City JEQUITIBA                     -0.2067512  0.1081013  -1.913 0.056547 .
tt:City JOANESIA                      -0.3536589  0.1081174  -3.271 0.001168 **
tt:City JOAO MONLEVADE                 0.1919792  0.1081094   1.776 0.076560 .
tt:City LAGOA FORMOSA                 -0.0595399  0.1082188  -0.550 0.582515
tt:City LIMEIRA DO OESTE              -0.2535561  0.1080875  -2.346 0.019492 *
tt:City MALACACHETA                   -0.1676120  0.1084231  -1.546 0.122951
tt:City MESQUITA                      -0.0109290  0.1082224  -0.101 0.919614
tt:City MINDURI                        0.0072944  0.1082596   0.067 0.946316
tt:City MONJOLOS                      -0.2523838  0.1082884  -2.331 0.020288 *
tt:City NOVA PONTE                     0.1328139  0.1081856   1.228 0.220331
tt:City PALMA                         -0.4755595  0.1081376  -4.398 1.42e-05 ***
tt:City PATOS DE MINAS                 0.5115029  0.1084210   4.718 3.34e-06 ***
tt:City PEDRA DO INDAIA               -0.2121598  0.1080920  -1.963 0.050394 .
tt:City PRESIDENTE JUSCELINO          -0.0188189  0.1082443  -0.174 0.862070
tt:City RIO PIRACICABA                -0.1681521  0.1081398  -1.555 0.120781
tt:City SANTO ANTONIO DO MONTE        -0.0090411  0.1082269  -0.084 0.933467
tt:City SAO GONCALO DO ABAETE         -0.1913689  0.1082234  -1.768 0.077809 .
tt:City SAO GONCALO DO RIO ABAIXO     -0.4071786  0.1081953  -3.763 0.000194 ***
tt:City SAO JOAO DO MANHUACU          -0.3553363  0.1084834  -3.275 0.001151 **
tt:City SAO JOSE DA SAFIRA            -0.4287740  0.1081281  -3.965 8.74e-05 ***
tt:City SAO SEBASTIAO DO RIO PRETO -0.3353637  0.1080866  -3.103 0.002059 **
tt:City SAO TOMAS DE AQUINO           -0.4083657  0.1082388  -3.773 0.000187 ***
tt:City SAO VICENTE DE MINAS          -0.1079862  0.1081335  -0.999 0.318599
tt:City SENHORA DOS REMEDIOS          -0.3979499  0.1081613  -3.679 0.000267 ***
tt:City UBERABA                        0.0306384  0.1082486   0.283 0.777301
tt:City VERISSIMO                     -0.0031269  0.1083135  -0.029 0.976984
---
Signif. codes:  0 '***' 0.001 '**' 0.01 '*' 0.05 '.' 0.1 ' ' 1

Residual standard error: 0.8016 on 384 degrees of freedom
Multiple R-squared:  0.8697,     Adjusted R-squared:  0.8398
F-statistic: 29.12 on 88 and 384 DF,  p-value: < 2.2e-16
```

Table S5. Full linear time-series regression model for DDD/1,000 inhabitants/year of analgesics and covariates.

```
> model3=lm(log(DDD)~ Coverage+FDA+Ext+Endo+DP+tt+City+tt:City,data=dt) #
> summary(model3)  # Adjust the least squares model.

Call:
lm(formula = log(DDD) ~ Coverage + FDA + Ext + Endo + DP + tt +
    City + tt:City, data = dt)

Residuals:
    Min      1Q  Median      3Q     Max
-3.8082 -0.3554 -0.0014  0.4504  3.5543

Coefficients:
                                    Estimate Std. Error  tvalue Pr(>|t|)
(Intercept)                         2.415e-01  6.628e-01   0.364 0.715757
Coverage                            1.983e-03  4.087e-03   0.485 0.627779
FDA                                 4.583e-05  6.140e-05   0.746 0.455837
Ext                                 6.965e-03  8.563e-03   0.813 0.416459
Endo                               -6.968e-03  8.563e-03  -0.814 0.416250
DP                                  2.352e-03  1.897e-03   1.240 0.215762
tt                                  2.775e-01  8.155e-02   3.404 0.000736 ***
City ARAPUA                         5.874e-01  7.812e-01   0.752 0.452537
City BALDIM                         2.343e+00  7.828e-01   2.993 0.002945 **
City BELA VISTA DE MINAS            2.716e+00  8.174e-01   3.323 0.000977 ***
City BONFINOPOLIS DE MINAS          2.854e+00  7.810e-01   3.654 0.000294 ***
City CARNEIRINHO                   -1.660e+00  7.850e-01  -2.115 0.035063 *
City CATAS ALTAS                    4.306e+00  7.961e-01   5.409 1.12e-07 ***
City CLAUDIO                       -1.287e-01  7.949e-01  -0.162 0.871425
City CONEGO MARINHO                 2.116e+00  8.809e-01   2.402 0.016772 *
City CONGONHAS DO NORTE             2.995e+00  8.026e-01   3.732 0.000219 ***
City CONQUISTA                      1.530e+00  7.824e-01   1.955 0.051314 .
City DOM BOSCO                      1.462e+00  7.829e-01   1.868 0.062550 .
City DOM CAVATI                     9.271e-01  7.815e-01   1.186 0.236238
City DOM SILVERIO                   5.312e-01  7.903e-01   0.672 0.501895
City DOURADOQUARA                   3.495e+00  7.885e-01   4.433 1.22e-05 ***
City INDIANOPOLIS                   3.622e+00  7.819e-01   4.633 4.96e-06 ***
City JABOTICATUBAS                 -3.023e+00  7.849e-01  -3.852 0.000138 ***
City JEQUITIBA                      7.442e-01  7.855e-01   0.947 0.344009
City JOANESIA                       2.654e+00  7.825e-01   3.392 0.000766 ***
City JOAO MONLEVADE                -3.824e+00  8.319e-01  -4.597 5.83e-06 ***
City LAGOA FORMOSA                  2.237e+00  7.970e-01   2.807 0.005263 **
City LIMEIRA DO OESTE               3.153e+00  7.813e-01   4.035 6.60e-05 ***
City MALACACHETA                    4.846e-01  7.852e-01   0.617 0.537484
City MESQUITA                       1.244e+00  7.820e-01   1.591 0.112536
City MINDURI                        4.509e-01  7.907e-01   0.570 0.568802
City MONJOLOS                       3.262e+00  8.103e-01   4.026 6.84e-05 ***
City NOVA PONTE                     8.839e-01  7.812e-01   1.132 0.258540
City PALMA                          3.876e+00  7.819e-01   4.957 1.08e-06 ***
City PATOS DE MINAS                -4.473e+00  7.969e-01  -5.613 3.83e-08 ***
City PEDRA DO INDAIA                3.289e+00  7.881e-01   4.173 3.73e-05 ***
City PRESIDENTE JUSCELINO           1.037e+00  7.811e-01   1.328 0.184917
City RIO PIRACICABA                -1.921e+00  8.247e-01  -2.329 0.020369 *
```

```
City SANTO ANTONIO DO MONTE        -3.062e+00  7.826e-01  -3.913 0.000108 ***
City SAO GONCALO DO ABAETE          1.669e+00  8.129e-01   2.053 0.040776 *
City SAO GONCALO DO RIO ABAIXO      4.311e+00  7.947e-01   5.425 1.03e-07 ***
City SAO JOAO DO MANHUACU           1.332e+00  7.814e-01   1.705 0.088962 .
City SAO JOSE DA SAFIRA             2.341e+00  7.866e-01   2.976 0.003106 **
City SAO SEBASTIAO DO RIO PRETO     3.832e+00  9.835e-01   3.896 0.000115 ***
City SAO TOMAS DE AQUINO            1.107e+00  7.817e-01   1.417 0.157433
City SAO VICENTE DE MINAS           2.049e+00  7.819e-01   2.620 0.009145 **
City SENHORA DOS REMEDIOS           2.914e+00  7.912e-01   3.683 0.000264 ***
City UBERABA                       -4.760e+00  7.851e-01  -6.063 3.21e-09 ***
City VERISSIMO                      2.227e+00  7.846e-01   2.838 0.004780 **
tt:City ARAPUA                     -1.401e-03  1.151e-01  -0.012 0.990291
tt:City BALDIM                     -1.557e-01  1.153e-01  -1.350 0.177700
tt:City BELA VISTA DE MINAS        -1.657e-01  1.162e-01  -1.426 0.154582
tt:City BONFINOPOLIS DE MINAS      -2.026e-01  1.152e-01  -1.759 0.079383 .
tt:City CARNEIRINHO                 2.515e-01  1.172e-01   2.146 0.032530 *
tt:City CATAS ALTAS                -3.480e-01  1.159e-01  -3.003 0.002850 **
tt:City CLAUDIO                    -3.085e-02  1.159e-01  -0.266 0.790187
tt:City CONEGO MARINHO             -2.863e-01  1.309e-01  -2.187 0.029364 *
tt:City CONGONHAS DO NORTE         -4.080e-01  1.168e-01  -3.492 0.000535 ***
tt:City CONQUISTA                  -4.236e-01  1.158e-01  -3.659 0.000289 ***
tt:City DOM BOSCO                  -2.982e-02  1.151e-01  -0.259 0.795763
tt:City DOM CAVATI                  7.358e-02  1.151e-01   0.639 0.522962
tt:City DOM SILVERIO                2.618e-04  1.201e-01   0.002 0.998263
tt:City DOURADOQUARA               -2.739e-01  1.156e-01  -2.369 0.018356 *
tt:City INDIANOPOLIS               -2.433e-01  1.154e-01  -2.109 0.035602 *
tt:City JABOTICATUBAS               2.167e-01  1.151e-01   1.882 0.060629 .
tt:City JEQUITIBA                  -2.155e-01  1.155e-01  -1.866 0.062797 .
tt:City JOANESIA                   -3.440e-01  1.151e-01  -2.990 0.002970 **
tt:City JOAO MONLEVADE              1.765e-01  1.152e-01   1.532 0.126349
tt:City LAGOA FORMOSA              -5.596e-02  1.184e-01  -0.472 0.636857
tt:City LIMEIRA DO OESTE           -2.485e-01  1.151e-01  -2.158 0.031513 *
tt:City MALACACHETA                -2.419e-01  1.154e-01  -2.096 0.036744 *
tt:City MESQUITA                   -4.245e-02  1.150e-01  -0.369 0.712252
tt:City MINDURI                    -2.773e-02  1.152e-01  -0.241 0.809889
tt:City MONJOLOS                   -1.937e-01  1.157e-01  -1.674 0.094868 .
tt:City NOVA PONTE                  1.102e-01  1.153e-01   0.956 0.339790
tt:City PALMA                      -4.955e-01  1.150e-01  -4.308 2.10e-05 ***
tt:City PATOS DE MINAS              4.532e-01  1.151e-01   3.939 9.74e-05 ***
tt:City PEDRA DO INDAIA            -2.058e-01  1.275e-01  -1.613 0.107502
tt:City PRESIDENTE JUSCELINO       -8.560e-02  1.170e-01  -0.732 0.464814
tt:City RIO PIRACICABA             -2.043e-01  1.198e-01  -1.706 0.088881 .
tt:City SANTO ANTONIO DO MONTE     -2.471e-02  1.151e-01  -0.215 0.830075
tt:City SAO GONCALO DO ABAETE      -2.340e-01  1.174e-01  -1.994 0.046907 *
tt:City SAO GONCALO DO RIO ABAIXO  -4.641e-01  1.221e-01  -3.802 0.000167 ***
tt:City SAO JOAO DO MANHUACU       -2.864e-01  1.151e-01  -2.489 0.013246 *
tt:City SAO JOSE DA SAFIRA         -4.315e-01  1.158e-01  -3.726 0.000224 ***
tt:City SAO SEBASTIAO DO RIO PRETO -2.865e-01  1.266e-01  -2.262 0.024256 *
tt:City SAO TOMAS DE AQUINO        -3.820e-01  1.150e-01  -3.321 0.000984 ***
tt:City SAO VICENTE DE MINAS       -1.374e-01  1.152e-01  -1.193 0.233737
tt:City SENHORA DOS REMEDIOS       -4.344e-01  1.163e-01  -3.734 0.000217 ***
tt:City UBERABA                     3.417e-03  1.152e-01   0.030 0.976363
tt:City VERISSIMO                  -3.253e-02  1.157e-01  -0.281 0.778736
---
Signif. codes:  0 ‘***’ 0.001 ‘**’ 0.01 ‘*’ 0.05 ‘.’ 0.1 ‘ ’ 1

Residual standard error: 0.8529 on 382 degrees of freedom
Multiple R-squared:  0.8532,          Adjusted R-squared:  0.8186
F-statistic: 24.67 on 90 and 382 DF,  p-value: < 2.2e-16
```
